# Methodologies and Challenges for Optimal Sensor Placement in Historical Masonry Buildings

**DOI:** 10.3390/s23239304

**Published:** 2023-11-21

**Authors:** Estefanía Chaves, Alberto Barontini, Nuno Mendes, Víctor Compán, Paulo B. Lourenço

**Affiliations:** 1 Department of Civil Engineering, ISISE, ARISE, University of Minho, 4800-058 Guimarães, Portugal; albe.barontini@gmail.com (A.B.); nunomendes@civil.uminho.pt (N.M.); pbl@civil.uminho.pt (P.B.L.); 2 Department of Building Structures and Geotechnical Engineering, University of Seville, 41012 Seville, Spain; compan@us.es

**Keywords:** optimal sensor placement, dynamic identification, structural health monitoring, heritage buildings, historical masonry

## Abstract

As ageing structures and infrastructures become a global concern, structural health monitoring (SHM) is seen as a crucial tool for their cost-effective maintenance. Promising results obtained for modern and conventional constructions suggested the application of SHM to historical masonry buildings as well. However, this presents peculiar shortcomings and open challenges. One of the most relevant aspects that deserve more research is the optimisation of the sensor placement to tackle well-known issues in ambient vibration testing for such buildings. The present paper focuses on the application of optimal sensor placement (OSP) strategies for dynamic identification in historical masonry buildings. While OSP techniques have been extensively studied in various structural contexts, their application in historical masonry buildings remains relatively limited. This paper discusses the challenges and opportunities of OSP in this specific context, analysing and discussing real-world examples, as well as a numerical benchmark application to illustrate its complexities. This article aims to shed light on the progress and issues associated with OSP in masonry historical buildings, providing a detailed problem formulation, identifying ongoing challenges and presenting promising solutions for future improvements.

## 1. Introduction

In recent decades, there has been a growing focus on and investment in tools and strategies for monitoring structural behaviour. This increased interest, especially in developed countries, stems from a significant number of critical facilities, infrastructures and buildings being close to or beyond the end of their design life. As their replacement is impractical and unaffordable, stakeholders increasingly turn to structural health monitoring (SHM) solutions to inform maintenance and restoration activities, optimising time and resource allocation. SHM arose as a field of research whose aim is to develop and validate methodologies and tools that provide information about the structure and its behaviour over time. SHM methods mainly focus on the prompt automated detection of damage, fostering interventions in the earliest stage possible and in the most effective way. This aims to avoid moderate and severe damage or even sudden failures and ensure proper operation and conservation of buildings [1,2,3].

The promising results obtained in the fields of mechanical, aerospace and civil engineering regarding modern structures suggested the application of SHM strategies to historical constructions as well [4]. Indeed, heritage buildings not only have a relevant economic value but also a significant social one due to their meaning for cultural roots, belief systems and individual identities [5]. Their conservation is, therefore, fundamental, but is commonly more challenging than for other types of civil structures due to their complexity in terms of geometry, materials and evolution over time. In particular, several uncertainties arise due to the lack of knowledge of the construction techniques employed, past interventions or hazardous events that they may have suffered [6].

Currently, purchasing and maintaining the required software and hardware components, including instrumentation, licenses, storage platforms and processing systems, require a significant investment. For these reasons, the development of a cost-effective SHM system is paramount. With this purpose, optimal sensor placement (OSP) emerges as a methodology that studies the optimisation of the sensor network, namely, the minimisation of the number of sensors used and the determination of their optimal locations, without compromising the effectiveness of the system. More specifically, the OSP is composed of eight main steps, as described in the framework developed by Ostachowicz et al. [7]: (1) definition of the demands, (2) choice of sensor type, (3) definition of the operational parameters, (4) determination of the objective function, (5) choice of the OSP technique, (6) definition of the inputs, (7) OSP resolution and (8) deployment.

Steps 1 to 6 consist of the definition of all the main aspects to set the specific problem instance and the OSP algorithm instance, whereas in the last two steps, namely, 7 and 8, the OSP is carried out and the network is deployed over the investigated system. More in detail, the definition of the scope of the monitoring (step 1) and the operational parameters (step 3), as well as the selection of the type of sensors (step 2), are essential to identify the expected use of the monitoring system, including the monitoring goals [8] and the final setup of the qualitative and quantitative problem-specific requirements. These can be related to the monitored system and its conditions, the budget available, the characteristics of the expected scenarios in case of damage detection, and the monitoring system and its components [7,8,9]. It is worth noting that points 2 and 3 are strongly interconnected, as the sensor type should be selected based not only on the demands but also on the operational parameters and, at the same time, the used sensors influence the definition of the operational parameters, which depend on the sensing network and its installation. In the field of OSP, most of the attention has been focused on vibration monitoring and dynamic identification [7]. Indeed, vibration monitoring through accelerometers or, more rarely, velocimeters, is a well-known global technique that is commonly adopted for structural identification and SHM, even in the field of historical buildings, since this technique provides general information about the system behaviour without causing any damage to it [1].

Several examples in the literature demonstrated the application of OSP techniques for the location of accelerometers in real structures, as in [10,11] or [12]. However, the use of these methodologies in historical buildings remains relatively limited. Even though there exist some review studies that analysed different OSP approaches, types of monitoring, metrics and optimisation algorithms [7,13,14], they mostly focused on civil structures and infrastructure. A careful analysis of the shortcomings that arise when established methods for conventional systems are applied to historical ones is missing. To this end, the present work aimed at bridging this gap, analysing the existing applications of OSP to historical buildings, or more specifically, OSP methods used to optimise the placement of transducers that are deployed in contact with the structure for dynamic identification, such as accelerometers, velocimeters or displacement transducers for output-only acquisitions. The methods discussed in the manuscript were originally developed to optimally place wired sensors; however, the same methods can be used for wireless sensors as long as additional requirements unique to this type of sensor are taken into consideration.

The aim was to identify successful adaptations to the built heritage of these techniques born in the context of mechanical, aerospace and modern civil engineering assets and to highlight the limitations met, pointing out progress made and open challenges, as well as identifying promising future trends for the development of successful enhanced and tailor-made strategies.

Several types of historical structures made of different materials and construction techniques exist. Some of them have been the subject of OSP. To ensure a clear analysis and synthesis of the results, the present work specifically focused on the application of OSP to historical buildings made of masonry, thus excluding applications to built heritage made of other materials, like timber [15,16] or concrete [17], and infrastructures such as metallic bridges [18], masonry bridges [19] and aqueducts [20]. Moreover, the scope of the OSP applications here investigated was limited to the determination of the optimal accelerometer placement for dynamic identification and, in a few cases, damage identification, with this being the objective of all the analysed studies.

The remainder of the paper is organised as follows. Section 2 details the definition and formulation of the OSP problem. Its application to masonry historical buildings is discussed in Section 3 based on the available literature, and a simple numerical case study is presented in Section 4 to better clarify the challenges. The main open issues are presented and analysed in Section 5 to propose promising strategies for future improvements. Finally, in Section 6, the main conclusions are drawn and future scopes are outlined.

## 2. Optimal Sensor Placement

The OSP can be formulated as a numerical optimisation problem in which one or more properly defined objective (or cost) functions $f_{obj}$ are maximised or minimised depending on the adopted metrics. In particular, considering that minimising $f_{obj}$ is equivalent to maximising $-f_{obj}$ [21], without loss of generality, the numerical optimisation problem is

(1) $$\underset{w\in \Omega }{\min}f_{obj,i}\left(w\right),\;(i=1,2,\ldots ,M)$$

subjected to:

$$h_{j}\left(w\right)=0,\;\left(j=1,2,\ldots ,J\right)$$

$$g_{k}\left(w\right)\leq 0,\;\left(k=1,2,\ldots ,K\right)$$

where $f_{obj,i}\left(w\right):D_{1}\times \ldots \times D_{n}\to R^{+}$ is the ith objective function in the problem; $D_{1}\times \ldots \times D_{n}$ is the search space given by the variable domain $\Omega \subseteq R^{n}$, namely, the region of feasible solutions in the search space; and $h_{j}\left(w\right)$ and $g_{k}\left(w\right)$ are constraint functions, namely, binary evaluations regarding specific requirements that the solution must satisfy to be feasible. All the functions depend on the design vector $w={\left(w_{1},w_{2},\ldots ,w_{n}\right)}^{T}\in \Omega$ whose components $w_{i}$ are called design or decision variables. Such variables can be continuous, discrete or a mixture of both. A feasible solution s is [22]

(2) $$s=\underset{D_{1}\times \ldots \times D_{n}}{\arg}\left(\underset{w\in \Omega }{\min}f_{obj,i}\left(w\right)\right),\;\;\;\;\;(i=1,2,\ldots ,M)$$

If M=1, the problem is called a single-objective problem, whereas if M>1, it is called a multi-objective problem. The best solution to the optimisation problem is the argument of the optimum for the objective function.

In OSP, there exist further limitations to the optimisation framework presented above. In particular, the only design variable is the set of locations and orientations, namely, the instrumented degrees of freedom (DOFs), which assume only discrete values and whose selection is mutually exclusive. This can be seen as a specialised Knapsack problem, namely, a constrained combinatorial problem in which a given number of sensors $n_{sens}$ are placed in a few locations and orientations among a larger set of candidates ($n_{cand}$), thus $1\leq n_{sens}\leq n_{cand}$ for a set of given objectives. A solution to this problem is a vector of spatial nodal coordinates $d=\left\{x_{1},y_{1},z_{1},\delta_{1},\ldots ,x_{n_{sens}},y_{n_{sens}},z_{n_{sens}},\delta_{n_{sens}}\;\right\}$, where $\delta_{i}=\left\{u_{i},v_{i},\;w_{i}\right\}$ is the orientation of the ith sensor and $\left\{x_{i},y_{i},\;z_{i}\right\}$ corresponds to its location in the reference system of the geometrical space. A simpler definition is achieved by resorting to a discretisation of the investigated system and the enumeration of the candidate DOFs: $d=\left\{d_{1},\delta_{1},\ldots ,d_{n_{sens}},\delta_{n_{sens}}\;\right\}\in D$.

The dimension of D, namely, the domain of the solutions, for a given number of sensors $n_{sens}$ and candidate locations $n_{cand}$ is

(3) $$\left|D\right|=\frac{n_{cand}!}{n_{sens}!\left(n_{cand}-n_{sens}\right)!}$$

In the literature, different criteria are commonly used to formulate the objective function and/or assess the suitability of the placement a posteriori [23]. To this end, the noteworthy work of Kammer [24] has been paramount, as it centred the focus on the formulation of objective functions, which ensures not only their observability but also their absolute identifiability, which is an essential requirement for correct dynamic identification. This has led to a proliferation of methods based on the Fisher information matrix (FIM), modal assurance criterion (MAC), singular-value decomposition ratio (SVDr), modal kinetic or strain energy (MKE and SE) or information-theory-related metrics [25,26,27,28]. These metrics are mainly based on the mode shape matrix partitioned at the candidate sensor locations and a pre-defined set of target modes. In any case, it is worth noting that the objective function is not known analytically and, consequently, the OSP is a black box optimisation problem. Moreover, the objective function is likely non-convex, presenting many local optima. Therefore, the objective function value at a specific point can be calculated only by running a simulation, and the traditional approach to OSP requires the existence of a preliminary numerical model, which is commonly a simplified finite element model (FEM), of the investigated system.

Addressing black box optimisation strongly limits the viable optimisation strategies [29]. These are mostly sub-optimal and, depending on their formulation, can be subdivided into deterministic or stochastic [7]. Deterministic methodologies treat the design variables as deterministic inputs. A wide range of methods have been developed to specifically address the optimisation of given metrics, leveraging ad hoc, non-generalisable strategies. These methods are the so-called heuristics, namely, a class of sequential sensor placement (SSP) algorithms, which iteratively add to or reject candidates from a predefined location set until finding the optimal solution [30]. Besides these methods, well-established techniques to tackle black-box optimisation, namely, the so-called metaheuristic algorithms, have been successfully applied to OSP. These are also sub-optimal methods but are not constrained by a specific formulation of the objective function, and thus, they allow for optimising more complex objective functions and consider some problem instances that are difficult to include in a heuristic process, such as multi-objective optimisation problems [7]. Multi-objective optimisation offers a powerful approach to simultaneously determining the optimal placement of sensors under multiple potentially conflicting goals, such as maximising coverage, minimising redundancy and controlling costs. Methods that are feasible for multi-objective optimisation produce a set of alternative solutions known as a Pareto front. Their comprehensive exploration of the solution domain, coupled with sensitivity analysis capabilities, provides a pivotal understanding of how different parameters and objectives influence optimal sensor configurations.

On the other hand, stochastic methodologies have been developed to treat the numerical parameters as random, considering the limited representability of the preliminary model adopted due to simplifications and limited knowledge of the physical and mechanical properties of the investigated structure. These methodologies may employ the aforementioned sub-optimal techniques within a probabilistic framework that repeats the optimisation upon sampling the values of the random variables from predefined distributions, ensuring an uncertainty analysis in the optimisation problem [7]. Instead, novel stochastic approaches are based on a formulation of OSP in the general framework of experimental design using a Bayesian approach [31], in which the goal is the maximisation of the value of the data collected through monitoring and the optimisation of time and cost requirements [32]. Finally, the necessity of dealing with several sources of uncertainties that affect not only the model but also the experimental stage of monitoring has recently led to novel approaches that circumvent the issues in the preliminary model by relying on a data-driven optimisation. Such approaches identify the best placement for the sensors among a large number of instrumented DOFs, resorting to preliminary monitoring, thus considering real fieldwork signals [33,34].

## 3. OSP Applications to Historical Masonry Buildings

**Figure 1:**
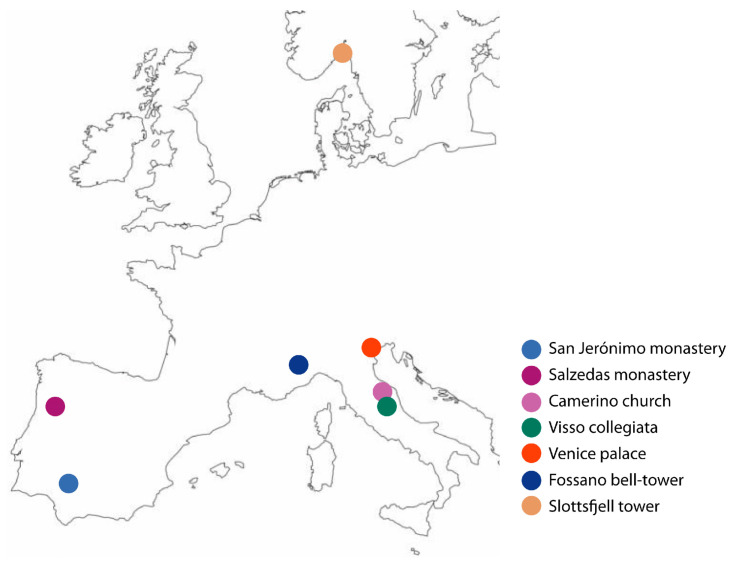
Location of the European case studies.

In the present section, a comprehensive analysis of the OSP applications to historical masonry buildings identified in the literature is provided, highlighting and comparing the main features of the optimisation process. The investigated case studies (Figure 1) comprise six religious buildings and two civil constructions, namely, the Santa Maria church in Via in Camerino, Italy [35]; the Collegiata of Santa Maria in Visso, Italy [36]; the bell tower of Santa Maria and San Giovenale Cathedral in Fossano, Italy [37]; the cloister of the monastery of San Jerónimo de Buenavista in Seville, Spain [38]; the cloister of the monastery of Santa Maria in Salzedas, Portugal [39]; the Cathedral of Saint John the Divine in New York [40]; the Slottsfjell tower in Tønsberg, Norway [19]; and a prototype of Venice palace inspired by Ca’ Loredan in Venice, Italy [41]. Notably, except for the monastery of Santa Maria in Salzedas, all the case studies employed a model-based OSP approach. Four cases optimised the placement for the complete building [19,35,36,41], whereas four focused on single portions, such as the bell tower [37], the cloisters [38,39] or a bay [40].

The objective of the optimisation process varies for different case studies, considering the type of building, level of damage (e.g., the Collegiata of Visso and Santa Maria in Camerino were significantly damaged by the Central Italy 2016 seismic sequence), previous interventions and maintenance activities (e.g., the extensive strengthening at the Salzedas monastery), the type of sensors (triaxial or uniaxial accelerometers), among other parameters. However, in general, the optimisation applied to the case studies aimed to efficiently design the sensor layout to ensure a correct modal identification with a reduced number of sensors and, consequently, improved cost effectiveness. The final sensors were natural candidates for long-term monitoring, thus allowing for the detection of a damage outbreak. To this end, it is worth noting that the OSP at the Fossano bell tower explicitly included damage identification by optimising the sensor placement in undamaged and expected damaged scenarios.

Regarding the selection of the candidates, the number and locations vary depending on the chosen approach. In the only data-driven application (i.e., the Salzedas monastery), the candidates corresponded to the monitored DOFs, which were a total of forty acquired DOFs evenly distributed over thirty-two points. In the case of the San Jerónimo monastery in Spain, an FEM was calibrated based on a preliminary acquisition and it was used for the OSP by considering the same candidate points as the dynamic campaign, namely, thirty-two points in three directions, summing up to ninety-six candidate DOFs.

**Figure 2:**
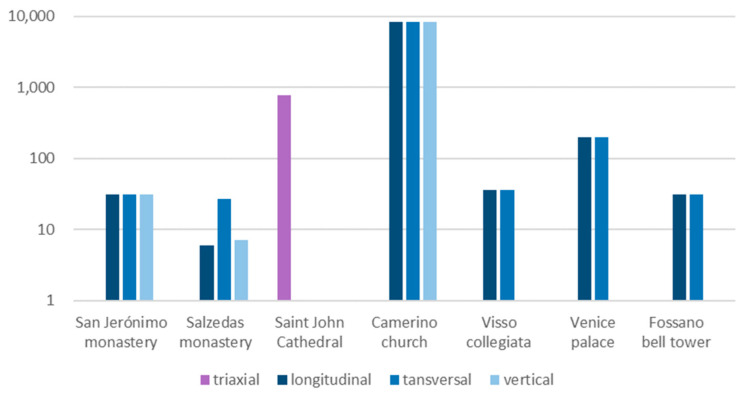
Number of candidate locations per direction for each case. Logarithmic scale. In Saint John Cathedral, the resultant of the three directions was used. No available information for the Slottsfjell tower.

For the other cases, the number of candidates is usually higher (Figure 2), as they are selected from the FEM itself. A common strategy to limit the candidates is based on the accessibility of the areas. For example, considering the application in the Camerino church, a random sampling approach was used to select candidate DOFs exclusively from the external surface. The initial set comprised over 250,000 DOFs, which were later refined to 25,000 through random sampling. This reduction served to reduce the computational burden while ensuring a representative subset for analysis. This approach was also applied to the Cathedral of Saint John, obtaining a total of 781 candidate positions from the complete set of accessible nodes of the FEM. The other applications followed a similar trend, selecting the candidates among the accessible DOFs of the FEM but limiting the number by manually excluding the areas with lower interest, or the vertical direction when vertical components of the mode shapes were deemed uninfluential or not targeted.

**Figure 3:**
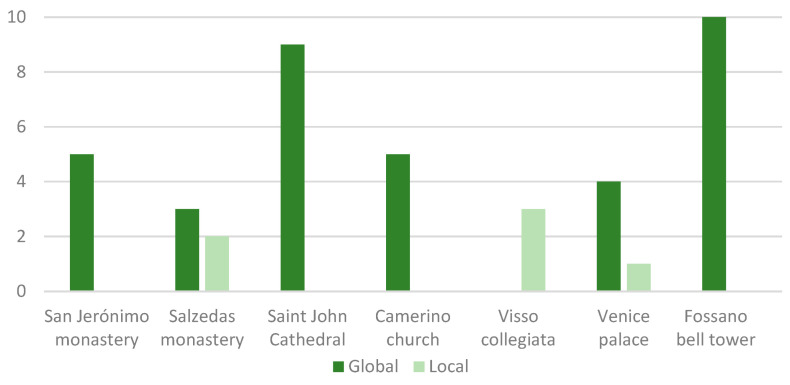
Number of global and local modes used in the optimisation in each case study.

Another important input for the optimisation is the number of target modes. It is interesting to note that most applications set this to five (Figure 3). In most cases, all of them represented global modes of the investigated building (i.e., the church of Santa Maria in Camerino and the Slottsfjell tower) or investigated building portions (i.e., the San Jerónimo monastery). The global modes of the church of Santa Maria in Camerino were two longitudinal, one transversal and two torsional. For the Slottsfjell tower, the first and the second modes were first bending, the third mode was torsional, and the fourth and fifth modes were second bending. At the Salzedas monastery, the presence of two local vertical modes of the slab of the investigated gallery of the cloister was addressed by carrying out one optimisation by considering the five identified modes, and another one considering just the three global modes, namely, two transversal, single and double bending modes, and a longitudinal mode. The optimisation conducted for the Venetian palace included one local mode of the internal courtyard within the five targeted. Here, the global modes were one longitudinal, one transversal, one torsional and a higher more complex global mode. In the Cathedral of Saint John, only the global modes (nine out of the first twenty) were considered. For the Fossano bell tower, 10 modes were used for the optimisation. In this case, the modes were local but involved the complete investigated macroelement. Similarly, at the Collegiata of Visso, the three targets were all local modes of the bell tower, two transverse in each direction and one torsional.

The algorithms used for most of the case studies were heuristics. The Fassano bell tower was the only exception by employing a metaheuristic algorithm. Most of the applications relied on the effective independence (EfI) method as the unique optimisation algorithm (i.e., Saint John Cathedral, Santa Maria church in Camerino, the Collegiata of Visso and the Venetian palace) or in comparison with alternative ones. This was the case of the Slottsfjell tower, where EfI was compared with the iterative Guyan reduction (IGR), the normalised modal displacement (NMD), the normalised kinetic (NKE) method and the off-of-diagonal MAC (offMAC). In the San Jerónimo monastery, the EfI was used and compared with the EfI weighted method (EfIwm), the kinetic energy and the strain energy matrix rank optimisation (KEMRO and SEMRO). Finally, for two cases, EfI was not considered. In the Salzedas monastery, five heuristic methods were used: eigenvector component product (ECP), mode shape summation plot (MSSP), average driving point residue (ADPR), weighted average driving point residue (WADPR) and QR decomposition (QRD).

For the Fassano bell tower, a metaheuristic approach was used, namely, a multi-objective genetic algorithm (MOGA) with cost functions based on MAC variations, i.e., both the auto-MAC, which compares the modes of a structure between themselves, and the cross-MAC, which compares the modes of two different structures or two different structural configurations of the same structure. In particular, the optimisation was carried out by considering two potentially conflicting goals upon damage onset: on the one hand, ensuring the identifiability of the modes for a given condition of the tower, and on the other hand, obtaining fairly distinct mode shapes in different conditions. The two combined objective functions guaranteed that the sensor pattern remains optimal throughout the lifetime of the structure, which is an important issue, especially for complex masonry buildings in high-hazard zones. In this study, the multi-objective method was compared with another metaheuristic technique, a single objective genetic algorithm (SOGA), as well as other heuristic methods: the entropy information (EI), ECP and ADPR methods.

When more than one optimisation algorithm is used, at least one validation metric is defined to compare the results from the different methods. For the Salzedas monastery, the comparison was conducted in terms of SVDr, the determinant of the FIM (detFIM) and the maximum offMAC value of the auto-MAC matrix. Moreover, the dynamic identification was carried out again, considering only the sensors recommended by the outperforming method, and the results were compared with the identification conducted with all the measurement points, validating the results through experimental data.

In the case of the San Jerónimo monastery, the comparison was carried out in terms of frequency error between the results obtained from the dynamic identification considering the complete set of candidates and the sensors obtained in the results. This is, indeed, the only model-based work that validates the results of the optimisation process based on experimental data. In the cases of the two towers where more than one method was used, the metric for the analysis of the final configuration was the offMAC value.

**Figure 4:**
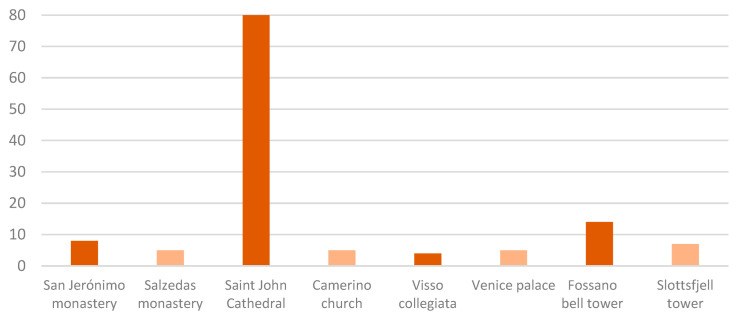
Number of sensors selected. Dark orange represents the cases where the number of sensors was optimised. Light orange represents the cases where the minimum number was pre-defined.

In most of the applications, as is common in the traditional OSP formulation, the number of sensors to be placed was predetermined, as shown in light orange in Figure 4. This was the case for the Slottsfjell tower, where seven uniaxial sensors were selected. Since for some algorithms, the minimum number of sensors to place is equal to the number of target modes, this is the pre-set value. This criterion was adopted in the Santa Maria church and the monastery in Salzedas. For the Venetian palace, the minimum number equal to the target modes (i.e., five) was first used, defining a set of so-called vital sensors. Then, configurations with an increasing number of sensors, up to ten, were considered. Developing a robust strategy to optimise the number of sensors is currently an open challenge. Some authors achieved this by selecting a range for the number of sensors and analysing the results by means of some performance metrics. This was the case for the Collegiata of Visso, where the sensors varied between three and six. The trace and the determinant of the FIM, the SVDr and the maximum offMAC value were used as performance metrics, and based on them, the best number of sensors was identified as four. For the Fassano bell tower, a minimum number of fourteen was defined as optimal without losing performance in terms of offMAC values. At the San Jerónimo monastery, the minimum number of sensors, between two and sixteen, was defined based on the analysis of the error in terms of frequencies between the identification using the data from the dynamic campaign and the identification using just the resultant sensors of the optimisation. The authors concluded that the error was stabilised for a number of sensors greater than eight.

In the case of the Cathedral of Saint John, a viability analysis of three potential metrics was conducted, namely, the loss of information measured by the determinant of the FIM, the MAC and the EfI value. It is important to note that the loss of information metric is relative and strictly depends on the initial candidate set’s characteristics. The MAC metric seems to be more suitable when dealing with a substantial number of sensors. As a result, the EfI value is favoured, and even though the primary objective of this method is not the definition of the minimum number of sensors, this metric is used for setting an arbitrary threshold of 0.1 to determine the number of sensors.

As can be seen in Figure 4, attempts to determine a minimum value for the number of sensors based on the metrics (cases highlighted in dark orange in the graph) generated very different results. This was likely affected by the problem input, such as the number and selection of target modes or the number and location of candidate nodes, as well as the choice of the metric itself. Moreover, it is potentially influenced by the way the adopted algorithm explored the domain of feasible solutions in the following iterations. It is therefore essential to define robust strategies to solve this open issue within the OSP.

A relevant issue of OSP for heritage buildings is the level of uncertainties involved in the definition of the model. In all cases, except one (i.e., the Venetian palace), the FEM used for the optimisation was updated based on dynamic identification tests to minimise this issue. In most cases, piezoelectric accelerometers were used for the identification, except for the San Jeronimo monastery, where force balance accelerometers were used. More details regarding the sensors adopted in these case studies are reported in Table 1.

In the Venetian palace, instead, the sources of uncertainty in the model were included in the optimisation using an extensive Monte Carlo simulation (MCS), treating several unknown features of the model and sampling 200 sets of values for them. Continuous distribution was used for the variables related to the walls, namely, elastic modulus and shear modulus with a log-normal probability distribution function (PDF) and mass density and thickness with a normal PDF. Discrete distributions were used for the floor properties and the connection between walls, considering three values with uniform probability in each case. Then, the optimisation was carried out for each sample, and the preliminary results were discussed without providing, at this stage, a strategy for the definition of the overall best placement.

A similar stochastic approach was used at the San Jerónimo monastery, where the MCS considered the elastic modulus of the brick and the stone masonries to be stochastic with normal distribution to generate the samples. For this case, the determination of the optimal number of samples was defined by analysing the dispersion percentage, which was stabilised for a number of samples greater than 64. In this case, the probability of sensor selection was computed for each method, obtaining the most recurrent positions.

A simpler approach to include uncertainties was presented for the Slottsfjell tower, where only two scenarios were analysed and compared: one where the tower was fixed to the soil, and a second one where the soil–structure interaction (SSI) was considered. Finally, the OSP application to the Cathedral of Saint John included the uncertainties according to an enhanced version of the EfI proposed in [42], which considered the possible inaccuracies of the numerical model by assuming a 5% error in the model input parameters. Thus, five new FEMs were generated with a 5% reduction in the elastic modulus of one of each material type at the time (assigned for ribs/arches, vault webbing, rubble surcharge, piers and walls) to be compared against a reference model.

## 4. OSP in Historical Masonry Buildings—Insight from a Simple Numerical Benchmark

To illustrate the aforementioned outstanding challenges, this section demonstrates the use of two main OSP techniques on a simple but effective benchmark, namely, the numerical model of a three-nave masonry church with a pitched roof, twin towers at the sides of the façade, a transept and an apse with two secondary chapels. The openings were asymmetrical in both the transept and in the towers to include more complexity. The overall dimensions of the three naves were 40 m by 20 m. A solid FEM with 243,000 elements was created with the software DIANA [43]. The elements were defined as quadratic/hexagonal mesh elements (primarily the CHX60 twenty-node isoparametric solid brick element).

The methodology proposed included an initial examination of eight different configurations according to a two-level full factorial design of the following three parameters (i.e., all combinations of parameters and levels were tested): candidate locations, number of sensors to place and heuristic optimisation algorithm adopted. One of these configurations was then chosen and compared with four other cases, where four alternative scenarios were simulated by modifying the model to account for uncertainties. The auto-MAC metric was then calculated for these four scenarios, using the chosen location configuration. Finally, one of the previous eight configurations was implemented in the four additional scenarios by considering a larger number of modes.

**Figure 5:**
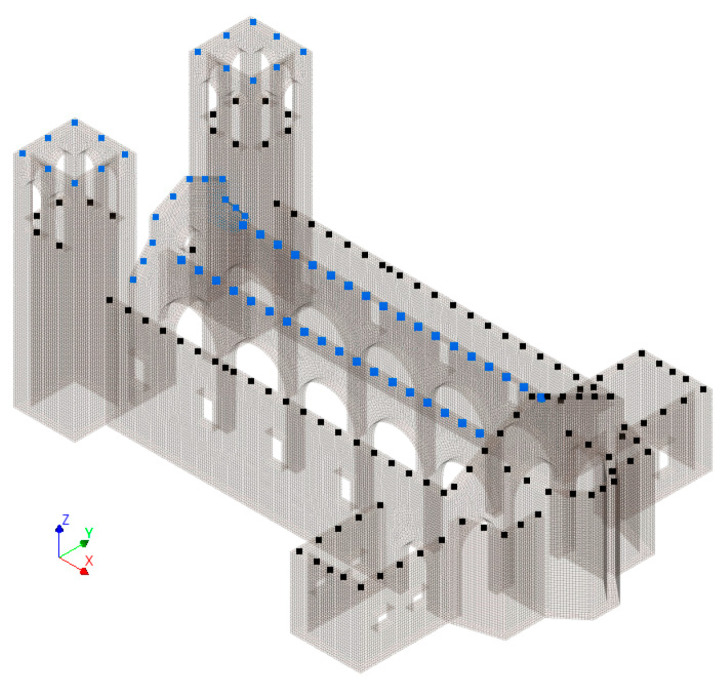
Candidate nodes (black: 113-node selection; black and blue: 179-node selection).

The reference scenario assumed that the longitudinal walls were interconnected by the roof, with all wall intersections perfectly interlocked. Within this reference scenario, two sets of candidate points were considered, as shown in Figure 5. One set consisted of 179 nodes, while the other was a selection of 113 nodes, which was achieved by eliminating areas that could pose accessibility problems in a real-world application, such as the upper level of the towers and the top of the central nave.

**Figure 6:**
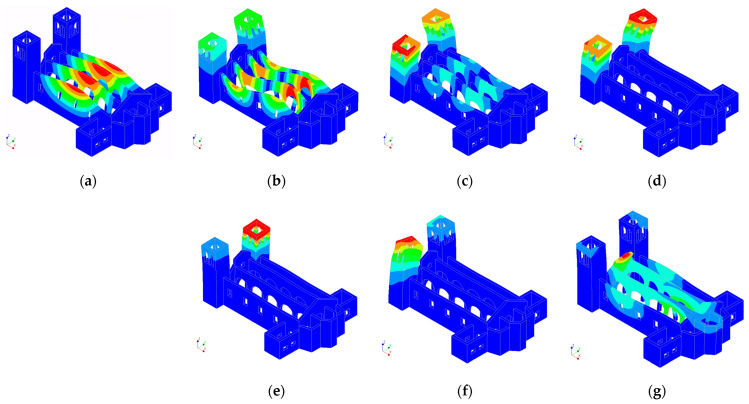
Modal shapes of the reference scenario used for the optimisation: (**a**) mode 1; (**b**) mode 2; (**c**) mode 3; (**d**) mode 4; (**e**) mode 5; (**f**) mode 6; (**g**) mode 9. Maximum displacemnt represented by red, zero displacement represented by blue.

For each node, the three translational DOFs were considered as candidates. Both sets of candidates were used for the optimisation with two well-established methods: EfI and the maximum offMAC methods. Finally, the final number of sensors to place was assumed equal to either 10 or 15, which was defined on the basis of the experience of the authors as a reasonable number considering the number of involved modes in the optimisation and the configurations of the other variations of the model analysed in this section. In this initial application, the seven modes shown in Figure 6 were employed, covering both the global modes and local modes of the towers. Modes 1 and 2 were mainly transversal modes of the naves, while modes 3 to 6 were local modes of the towers. Modes 7 and 8 affected the central nave but they were rejected for their complexity. Finally, mode 9, which represented a longitudinal mode of the nave, was selected.

**Figure 7:**
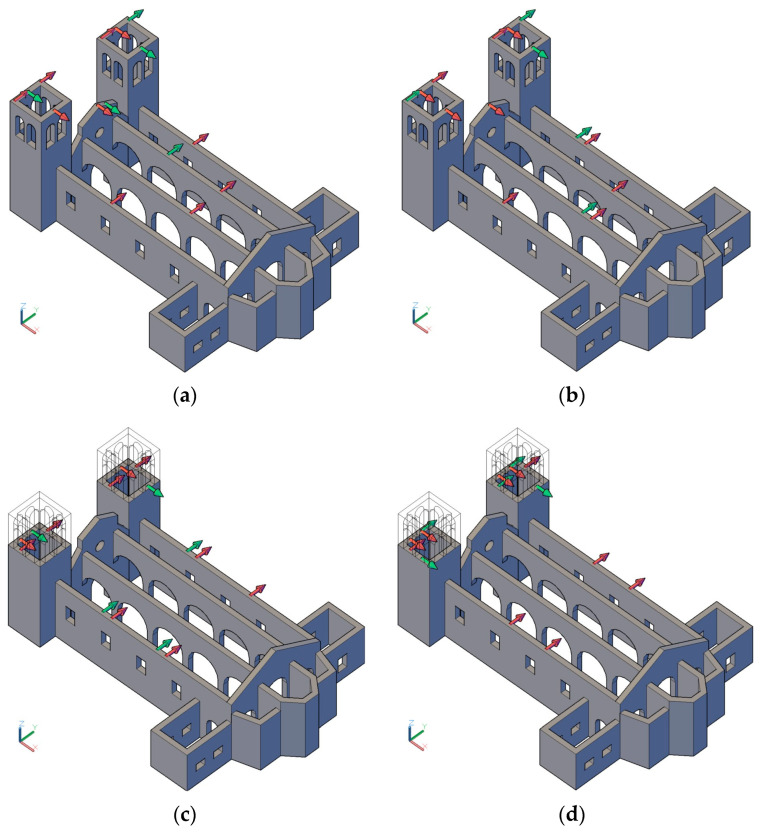
OSP results. Outcomes of the 10-sensor optimisation are shown with red arrows, with the five additional sensors (15-sensor optimisation) in green: (**a**) 179-node EfI method; (**b**) 179-node offMAC method; (**c**) 113-node EfI method; (**d**) 113-node offMAC method.

For the results of the 179-node case (Figure 7a,b), both methods yielded similar placements when 10 sensors were selected. In this arrangement, one sensor was placed on each of the longitudinal walls in the transverse direction; one on the façade along the longitudinal direction; and five sensors were distributed along the towers, covering both horizontal directions. However, when five additional sensors were included, a clustering phenomenon appeared. In this case, three of the additional sensors were located at the top of the towers, and each tower had two sensors in both horizontal directions. When the offMAC-based optimisation method was used, the two supplementary sensors were located on the nave, close to two of the existing sensors (Figure 7b). With the EfI method, the clustering appeared in the façade, but not in the naves (Figure 7a).

When 113 candidate nodes were considered, the 10-sensor optimisation provided a configuration that closely resembled the equivalent one obtained from the 179 candidate nodes, with minimum adjustments due to the reduction in the feasible solutions. For this reason, the sensors placed in the tower moved from the upper to the lower level, and the sensors placed on top of the central nave moved to the corresponding positions on the external walls. It is worth noting that considering the top of the façade was inaccessible, the sensor that was intended to be located here was moved to the tower and the final configuration lacked any sensor monitoring the nave in the longitudinal direction. When 15 sensors were employed, the clustering problem became even more pronounced due to limitations on the number of candidates, as can be observed in Figure 7c in the nave and Figure 7d in the towers.

For a further analysis of the results obtained using the OSP methods, the layout obtained was compared with the placement based on expert judgement in similar case studies, namely, Saint Torcato church [44], Matera Cathedral [45], church of the Monastery of Saint Miguel de Refojos [46] and church of Saint Juan Bautista de Huaro [47]. It is worth noting that in all these cases, the number of measured DOFs was larger than in the numerical benchmark, thus only a qualitative comparison can be conducted.

In the Saint Torcato church, sensors were distributed symmetrically in the towers. This happened in the benchmark 179-node 15-sensor case but not in the other cases. In Matera Cathedral, seven uniaxial accelerometers were located in the corners of the tower. The sensors were distributed over two levels and two horizontal directions. The results for the benchmark case (179-node) only located sensors in the higher level of the towers and not always in the corners; however, as in Matera Cathedral, the locations were distributed in both horizontal directions.

Regarding the nave, for the three-nave Matera Cathedral, the one-nave church of the Monastery of Saint Miguel de Refojos and the one-nave church of Saint Juan Bautista de Huaro, the accelerometers were always located on the external walls measuring the corners in both horizontal directions and distributing two (at Matera and Saint Miguel de Refojos) or three (at Huaro) accelerometers along the transversal direction over the longitudinal walls. In the benchmark, two sensors in the transversal direction of both lateral walls were included in the 113-node scenarios only.

Four additional scenarios were created to account for reasonably expected uncertainties within the model due to limited knowledge of the building. Scenario SCN01 assumed a poor connection between the top of the longitudinal walls (roof without stiffness and/or weak connection to the masonry walls). The following three scenarios included the same poor connection plus a disconnection between the nave and the towers (SCN02), the presence of soft soil (SCN03) and a disconnection of the transept from the nave (SCN04). Soil settlement is a common threat to historical massive buildings especially churches, whereas disconnections are not uncommon in such constructions that have often undergone several alterations with additions of portions over time.

**Figure 8:**
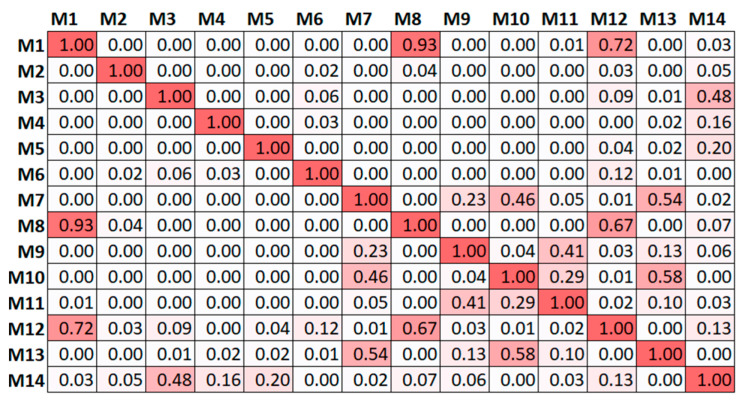
Auto-MAC matrix of the reference scenario, considering all the modes up to 5 Hz and the EfI 10-sensor 179-node optimisation. MAC values represented by colour scale and number.

First, the capability of the optimised placement based on the initial assumptions, through the model of the reference scenario, to correctly interpret the modes of the building in the presence of one of the alternative scenarios was tested. To this end, the final locations from the original 179 candidates for the 10 sensors provided using the EfI method were used to simulate monitoring by partitioning the mode shape matrix to the measurement DOFs and all the modes in the range up to 5 Hz. Since the modes selected for the optimisation of the reference model were not representative of the alternative scenarios, the latter criterion was established by considering a bandwidth that was likely to be excited by ambient sources of vibrations, and thus, are commonly identified during field tests. Applying this criterion to the reference scenario, 14 modes were found. The auto-MAC for these modes, considering the 10 measurement points, is shown in Figure 8.

**Figure 9:**
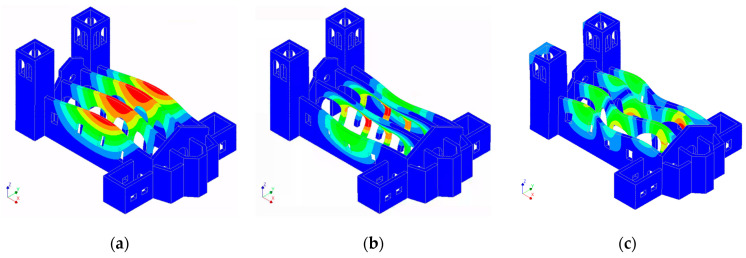
Modal shapes of the reference scenario: (**a**) mode 1; (**b**) mode 8; (**c**) mode 12. Maximum displacemnt represented by red, zero displacement represented by blue.

The modes used for optimisation (i.e., modes 1 through 6 and mode 9) appeared to be clearly distinct, with the maximum off-diagonal value reaching 0.23. However, distinguishing between mode 1 and mode 8 was complicated with the current sensor configuration, as the MAC value was 0.93. Similarly, modes 1 and 12 presented a MAC value of 0.72. In Figure 9, these modes can be compared visually. This highlights the critical role of mode selection during optimisation, even when the preliminary model is perfectly representative of the real scenario, as modes that are not targeted during the optimisation may be less identifiable or interpretable through the reduction in measurement points.

**Figure 10:**
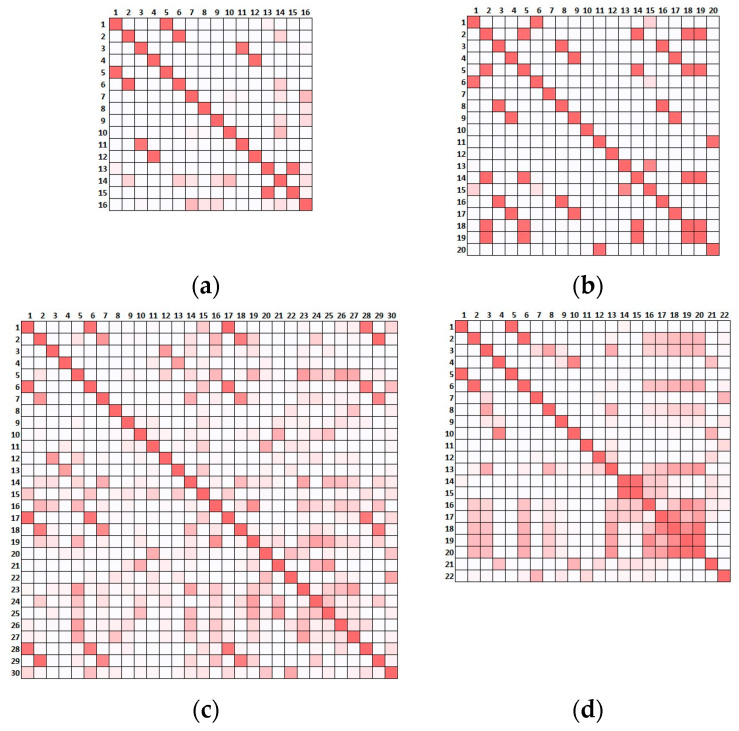
Auto-MAC tables considering all the modes under 5 Hz and the EfI 10-sensors 179-node optimisation for the reference scenario: (**a**) SCN01; (**b**) SCN02; (**c**) SCN03; (**d**) SCN04. MAC values represented by colour scale: red represents 1, white represents 0.

In what concerns the alternative scenarios, local modes appeared due to the reduced connection between the distinct portions of the buildings leading to 16, 20, 30 and 22 modes under 5 Hz for SCN01, SCN02, SCN03 and SCN04, respectively. The auto-MAC calculated for the alternative scenarios, considering such modes and the 10 sensors, is reported in Figure 10. Here, for the sake of clarity, the values are not presented but only the colour scale is shown and the higher the number and intensity of the red squares, the more difficult it was to differentiate and identify the modes.

Comparing the results of Figure 10 with Figure 8, there was a clear deterioration of the performance of the optimised placement when the actual scenario presented slight alterations with respect to the original assumption used for the OSP. This implies that whenever a limited level of knowledge leads to a preliminary model that is not representative of the real building, the optimisation conducted on a deterministic model can easily fail to distinguish mode shapes activated by overlooked features of the real structural configuration. The presence of a poorer connection at the roof level (SCN01) led to at least five couples of modes being almost indistinguishable (Figure 10a). The disconnection in the tower (SCN02), the presence of soft soil (SCN03) and the disconnection of the transept (SCN04), dramatically increased the couples of similar mode shapes, as presented in Figure 10b–d, respectively.

**Figure 11:**
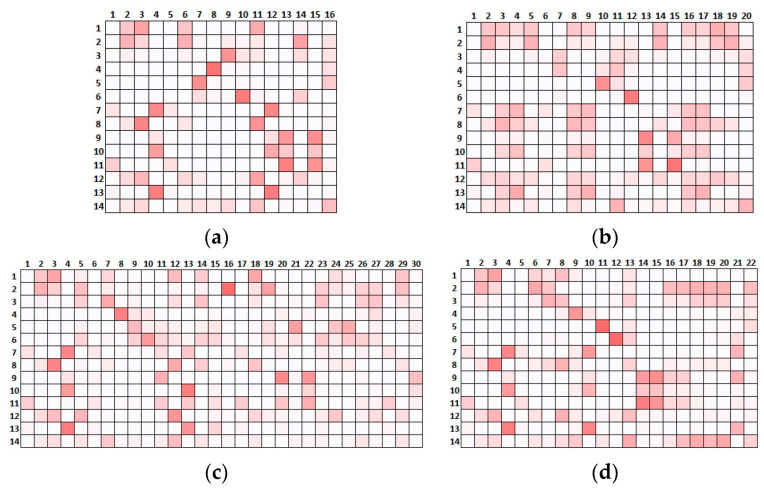
Cross-MAC tables. Comparison between the reference scenario and (**a**) SCN01, (**b**) SCN02, (**c**) SCN03 and (**d**) SCN04. MAC values represented by colour scale: red represents 1, white represents 0.

Consequently, one can ask whether the optimised placement was at least capable of informing the structural identification through the extracted modal properties, namely, whether the information provided by the identified mode shapes was sufficient to distinguish the actual scenarios. Although this is a complex task that in real applications requires an interpretation of the experimental data and a detailed model calibration, a simple test was conducted here by evaluating the cross-MAC, namely, the MAC between the reference scenario and the four alternative ones (Figure 11). The intensity of the red scale suggests that several modes in the pairwise comparison of the scenarios may be confused, potentially preventing a profitable use of the extracted shapes to calibrate the model and support the identification of the actual scenario.

**Figure 12:**
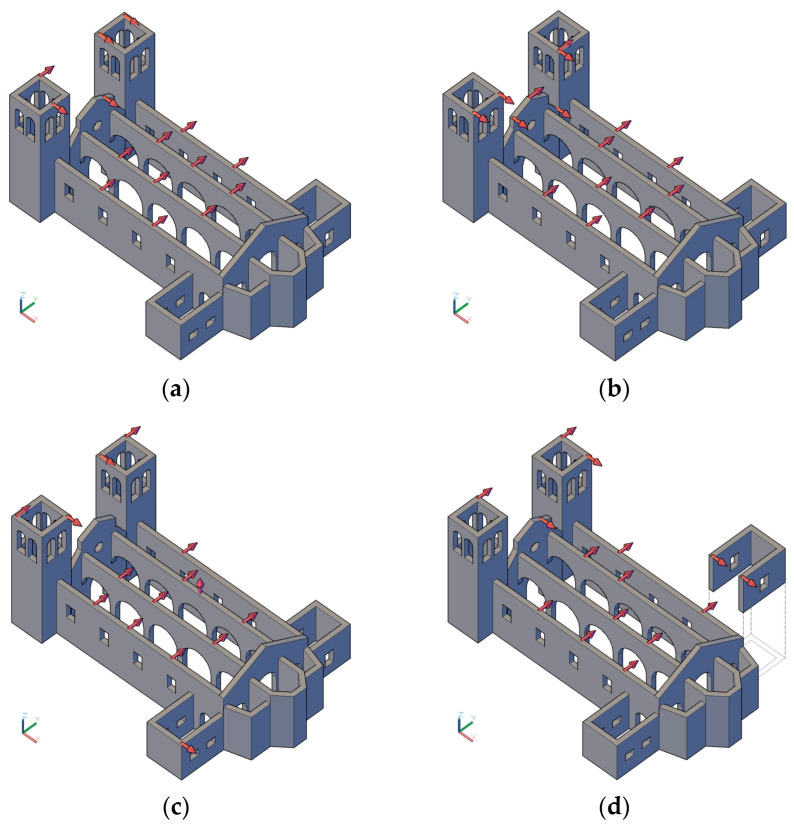
OSP results of the alternative scenarios: (**a**) SCN01; (**b**) SCN02; (**c**) SCN03; (**d**) SCN04.

Finally, optimisation was conducted on the models of the alternative scenarios to analyse the differences in the final placements. The results of the optimisation are illustrated in Figure 12. In this optimisation, the selected candidate nodes were the complete initial 179 nodes, leading to 537 DOFs for the uniaxial sensors. The number of modes varied for each scenario, comprising all global and local ones in the range up to 5 Hz.

The number of sensors placed was 15 and the optimisation method was based on the offMAC method. This approach was preferred since for the EfI, the minimum number of sensors cannot be less than the number of modes, which in these scenarios, would exceed 15 and be variable, preventing a fair comparison.

In general, the sensors were located and distributed throughout the building without exhibiting clustering. This could be attributed to the inclusion of a broader range of modes in the optimisation and was particularly evident for the towers. As the number of local modes of the towers was balanced by a larger number of modes involving the other components, their role became less relevant, leading to a reduction in the number of sensors placed there.

The rest of the sensors were distributed mainly along the length of the building. The façade contained one sensor in SCN01 and SCN04, but, as expected, it gained importance when it was disconnected from the towers in SCN02, containing more sensors in the final placement. The only case where a vertical sensor was introduced was in the scenario representing the soft soil and was located in the centre of one of the arcades. This was likely due to the way the soil–structure interaction was modelled, allowing for the occurrence of vertical modal displacement. Finally, SCN04 incorporated two sensors in a side chapel at the disconnection point. Due to the poorer involvement of the apse in the mode shapes, no sensors were placed in this area in any of the scenarios.

**Figure 13:**
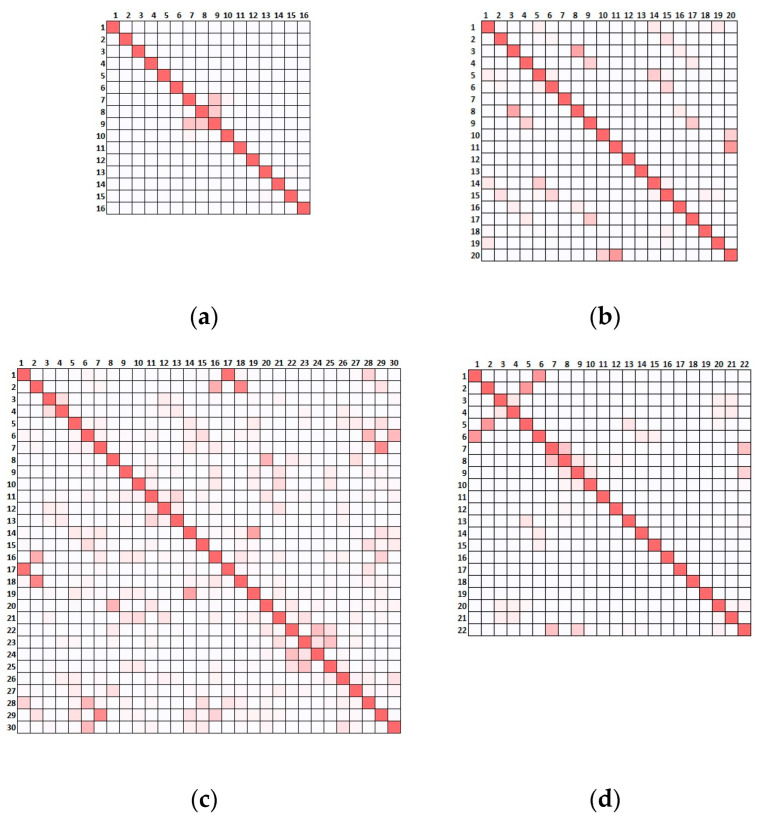
Auto-MAC tables considering the result from the offMAC 15-sensor 179-node optimisation of (**a**) SCN01, (**b**) SCN02, (**c**) SCN03 and (**d**) SCN04. MAC values represented by colour scale: red represents 1, white represents 0.

The auto-MAC was calculated for all the results (Figure 13). When comparing these outcomes with Figure 10, the improvement in the identifiability of the modes was evident, as these were targeted in the optimisation, resulting in less indistinguishable couples of modes.

## 5. Main Open Issues in OSP for Historical Masonry Buildings

Upon the comprehensive analysis of the literature and the application to the numerical benchmark discussed in the previous section, a clear set of relevant open issues and challenges that hinder a successful application of OSP methodologies to historical masonry buildings were identified and hereafter discussed. Such challenges can be classified according to the three main components of the OSP strategy: model-related, experiment-related and optimisation-related challenges.

Commonly, the OSP problem is addressed before a detailed investigation of the structure, with the goal being the identification of the best measurement points to maximise the information quality and minimise the resources needed for the acquisition. To this end, a preliminary model that is sufficiently representative of the structure is used to predict the expected behaviour. The generation of a reliable model involves the definition of numerous parameters that usually present a high level of uncertainty [48]. Such parameters comprise material properties, complex geometries, connection between elements, presence of damage and soil structure interaction, among others [19]. To minimise the uncertainties related to geometry, advanced surveying techniques, such as photogrammetry or laser scanning, are commonly employed. These tools can provide detailed values of deformations and damage locations, as well as accurate information for the definition of the structural numerical model. However, avoiding uncertainties in the definition of the other parameters is not so simple. One of the fundamental challenges in modelling historical masonry structures is the accurate characterisation of the material properties of aged and weathered masonry [49]. Traditional construction materials often lack the homogeneity and consistency seen in modern materials, making it difficult to define reliable values for the analysis. The construction techniques employed in historical masonry buildings can vary significantly from contemporary practices. These techniques may not be well documented and understanding them is crucial for creating accurate models. Additionally, historical masonry structures have often undergone modifications and repairs over the years. Documenting these alterations and understanding their impact on structural behaviour is essential but cumbersome. Numerical models should be able to accurately account for these changes. Determining the load history of a masonry structure is also challenging, especially when considering long-term effects, such as settlement, creep and thermal cycling [50]. Obtaining accurate load data for historical structures is often impossible, and assumptions need to be made, which can introduce uncertainties into the numerical model.

This kind of uncertainty undoubtedly compromises the quality of the results obtained using the OSP methodologies if a purely deterministic approach is followed. As shown in the benchmark application, slight changes to the original assumptions and unexpected configurations are hardly identifiable and distinguishable when their main features are not accounted for in the preliminary model used for the OSP. Similarly, including the uncertainties in the optimisation, as demonstrated by several applications (i.e., Venice palace, San Jerónimo monastery, Slottfjell tower and the numerical benchmark), leads to distinct optimised placements, calling for the definition of a robust strategy to select a final overall optimal localisation. The deterministic applications in literature try to circumvent the problem by employing a numerical model calibrated to the experimental response of the building. However, this solution requires a preliminary acquisition, failing to fulfil the original premise of providing an optimised placement before conducting any test. On the other hand, stochastic approaches try to include uncertainty analysis in the optimisation problem by simulating a wide range of possible scenarios. Two of the investigated applications proposed the MCS technique to sample several different instances from pre-set probabilistic functions for each stochastic variable [21,22]. However, this approach is much more computationally demanding, and several aspects are not fully agreed upon and need more research, such as the definition of the number of samples, the variables and their statistical distributions, and the final configuration considering the dispersion of the results.

Data-driven methods constitute a quite novel and completely alternative approach to avoid problems with uncertainties related to the definition of the model. In this case, the aforementioned premise of OSP is completely subverted and the aim becomes the determination of the optimal placement starting from a large number of measurement points recorded during an extensive ambient vibration test. This approach, indeed, opens a new scenario, in which the stakeholders prefer to invest in a large testing programme and a very high level of knowledge of the structure is obtained, making the generation of the numerical model unnecessary for the optimisation purpose. This implies avoiding the costs of creating a reliable numerical model, the complexity of dealing with the uncertainties, and the need for the necessary skill and expertise for the numerical simulation. On the other hand, this approach presents clear shortcomings, such as the cost of conducting a large testing programme in advance that for its scale, may interfere with the regular functioning of the building. Moreover, the lack of previous information about the dynamic behaviour of the structure may hinder proper test planning. A discussion of these two alternative scenarios (i.e., data-driven, high level of knowledge and experimentally intensive without numerical simulations required vs. model-based, low level of knowledge and computationally intensive without preliminary experiments) has been provided in [34].

The analysis of the data-driven approach leads to the discussion on experiment-related issues [39]. Such issues affect the campaign conducted before the application of data-driven OSP but also the preliminary test for numerical calibration purposes in model-based approaches [38]. Moreover, such issues should be taken into account while conducting the optimisation, irrespective of the adopted approach, to enhance the optimised monitoring.

Historical masonry buildings possess unique characteristics that greatly impact the modal analysis process [51]. These structures are often massive and exhibit potentially nonlinear responses and complex damping mechanisms due to ageing, material degradation and structural modifications, making them inherently different from more modern, homogeneous constructions. Furthermore, they can exhibit brittle behaviour, and their complex layouts, as seen in structures like churches, defy the simplifying assumption of box-like behaviour.

The challenges presented by these peculiarities extend to the domain of experimental modal analysis [52]. Historical masonry building high and local modes are not easily excited, and thus, can be hardly identified. However, the very essence of these structures calls for an optimisation of the sensor placement driven not only by the global modes but also by the local ones, which are often overlooked due to the complexity they introduce but play a key role in the structural response. A homogeneous selection of modes from different components and all major directions is also essential to avoid biases that arise from a selection that prioritises certain macroelements or mode shapes. All these issues can be observed in the application of the benchmark. Since all these decisions significantly influence the final configuration, it is important to define a global strategy that can be extrapolated to other cases of masonry buildings, despite the case-specific peculiarities.

In light of these structural complexities, there are several other pressing issues to contend with. Ambient vibration sources in the vicinity of such historical buildings may be weak, exacerbating the challenges in acquiring reliable modal data. These buildings often stand in restricted areas or, in general, far from heavily trafficked areas, where ambient vibrations are typically more substantial. Moreover, the operations around these heritage structures may introduce non-stationary inputs, including the periodic tolling of bells, micro-tremors and other sporadic disturbances. Traditional output-only modal analysis techniques, which are primarily designed for stationary and time-invariant inputs, struggle to handle these variable forces effectively [52].

Practical considerations are equally significant. Historical masonry buildings often operate within constraints imposed by their significance. This means that the installation and operation of the sensing devices must be minimally invasive and visually unobtrusive to comply with the conservation principles of these cultural assets, namely, preserving their visual and structural integrity. This limits, in many cases, the feasible candidate locations. However, selecting a sufficient number of candidate points to ensure the adequate performance of the optimisation strategy is paramount [39].

Not only the input of the optimisation process poses a problem but also the optimisation algorithm itself. Regarding the optimisation-related issues, the importance of the selection of the technique was demonstrated, which is a complex decision because of the large number of available alternatives, with all of them having different advantages and disadvantages. The applications in the literature that compare several optimisation methods, as in the case of the monastery in Salzedas, concluded that quite large variability in the estimation of the relevance of each candidate according to distinct metrics emerged, especially when the global and local modes were targeted.

In the case of the San Jerónimo monastery, among the four OSP algorithms analysed, the EfI method provided a solution that allowed for the identification of natural frequencies with less error, whereas the solution of the KEMRO method was the one that gave the greatest error in the modal identification. When uncertainties were considered for this structure, the SEMRO method was the one that presented the lowest dispersion in its solution for all the scenarios analysed. The different results and conclusions exposed the lack of robustness in OSP, as different methods provided very distinct placements and presented significant variability in the performance for various scenarios and investigated cases.

For the Fassano bell tower, multi-objective optimisation was addressed using genetic algorithms. In this case, the metrics were based on MAC functions. In particular, two objective functions were combined to ensure that the sensor pattern remained optimal throughout the lifetime of the structure, allowing for the successful detection of damage onset reflected in the alteration of the mode shapes. Although this approach offers several advantages, it requires the definition of more input parameters and the selection of the final single optimal solution is challenging, as a trade-off between potentially competing objectives must be found, which involves choosing among Pareto-optimal solutions that offer varying advantages in distinct aspects.

An additional inference to draw pertains to the necessity of establishing a methodology for determining the optimal number of sensors to place. In most cases, no optimisation was conducted, and the number was predefined based on specific demands, such as the availability of the sensors or a limitation of the budget. Indeed, the problem of optimising the network cost, possibly considering purchasing and installation costs for different types of sensors (tri-, bi- and uniaxial), even in combination, and the ease of access of the measurement points have not attracted much attention yet [37]. Thus, a recurrent strategy to determine the number of sensors to place was to set it equal to the number of targeted modes. More complex approaches analyse the evolution of one or more metrics for a variable number of sensors [38]. However, by lacking a benchmark for the minimum necessary value of these metrics, the identification of their optimum can provide an unrealistic number of sensors in complex cases, generating redundant information. This latter problem often emerges, even for a rather limited number of sensors, due to the tendency of several algorithms to cluster them. Indeed, as demonstrated by the benchmark application but also by the investigated literature (i.e., monastery of Salzedas and Venice palace), the adopted metrics often reward the point with larger modal displacement, whereas they tend to penalise nodes of the modes that would often be equally important for the interpretation of the shapes with more inflection points. As shown in the benchmark, this problem also depends on the number of modes chosen for optimisation, and thus, a wider selection of local and global modes and their proportion relative to the number of sensors could minimise this problem. In the Cathedral of Saint John, to address this issue, the EfI method was improved, including a distance-based criterion (DBC) to reject sensors closer than a minimum distance. Although the authors analysed various minimum distances from 0 to 3 m and the method provided promising results, it required a case-specific definition of the DBC that is hardly generalisable and applicable to other cases.

## 6. Conclusions

In the present paper, a comprehensive overview of OSP methods for historical masonry buildings is presented, which involves an introduction to the OSP problem, including an explanation of the involved steps and problem formulation. The paper comprises a review of eight applications found in the literature. The examined case studies encompass six religious buildings and two civil constructions. Four cases optimised the placement for the complete structure, while four focused on specific portions of it. The analysis covered objectives, input definition, candidate and mode selection, optimisation algorithms, result validation metrics, minimum sensor selection and uncertainty incorporation, aiming at identifying the unresolved issues associated with applying this technique to historical masonry buildings.

Based on these identified issues, a numerical benchmark was generated to illustrate the challenges more effectively. Two methods, namely, EfI and offMAC, were utilised, comparing various initial candidate locations and sensor quantities. Additionally, five scenarios (i.e., one reference and four alternatives) were defined through slight variations in the numerical model to account for typically expected uncertainties due to limited knowledge of the investigated historical building.

The analysis of the eight papers and the results obtained from the numerical benchmark yielded significant insights into the challenges associated with OSP in historical masonry buildings. These challenges could be categorised into three primary aspects: model-related, experiment-related, and optimisation-related challenges:

- Model-related problems stem from the unique characteristics of these buildings and the alterations that likely affected them over time, making the modelling task extremely challenging and affected by significant sources of uncertainty, which, if not properly addressed, may lead to a simulated behaviour not being representative of the real one.
- Experiment-related problems originate from common peculiarities of the historical masonry buildings that affect the interpretation of ambient vibration test data, such as the brittle behaviour and nonlinear response, the difficulty in exciting high and local modes despite their relevance, and the availability of mostly weak vibration sources with non-stationary inputs. Moreover, conservation principles prevent the adoption of invasive and obtrusive sensor installations.
- Optimisation-related issues depend on the optimisation algorithms and procedures, as existing methodologies fail to provide a univocal answer to the main goals of OSP. Variability in the optimised locations for the same application through distinct methods is significant and robust strategies to optimise the number and the overall costs along with the placement are still missing.

Building on the insights gained from this study, future research in the field of OSP for historical masonry buildings should focus on addressing the identified challenges and exploring novel techniques for optimising the sensor placement while considering the unique characteristics of the historical masonry structures. One potential avenue for further investigation is the development of cost-effective techniques that account for the uncertainties within the optimisation. Additionally, research could delve into innovative OSP methods to include costs and budget-related aspects. In this respect, one of the major problems consists of the selection of an optimal minimum number of sensors. To this end, multi-objective optimisation strategies could offer some advantages by allowing for managing several potentially conflicting objectives at the same time.

Finally, it is important to mention the need to explore issues related to experimentation, such as the difficulties in experimentally exciting and identifying specific modes and defining viable solutions to account for them within the optimisation process.

## Figures and Tables

**Table 1:** Characteristics of the sensors adopted for the dynamic identification of the investigated case studies.

Case	Type	Axis	Model	Sensitivity	Accel. *	Points **
San Jerónimo monastery	Force balance	Uni	KINEMETRICS ES-U2	10 V/g	8	32
Salzedas monastery	Piezoelectric	Uni	PCB 393B12	10 V/g	12	40
Saint John Cathedral	Piezoelectric	Uni	PCB 393A03	1 V/g	7	7
Camerino church	Piezoelectric	Uni	PCB 393B31	10 V/g	16	28
Visso collegiata	Piezoelectric	Tri	-	1 V/g	4	11
Fossano bell tower	Piezoelectric	Uni	PCB 3701G3FA3G	1 V/g	20	20

* Number of accelerometers used, ** number of positions measured.

## Data Availability

The data are available on request.
